# Simvastatin Restores Down-Regulated GATA-6 Expression in Pulmonary Hypertensive Rats

**DOI:** 10.1080/01902140902736819

**Published:** 2009-06-09

**Authors:** Bin Liu, Xiao-qin Wang, Li Yu, Tong-fu Zhou, Xian-min Wang, Han-min Liu

**Affiliations:** Department of Pediatric Cardiology, West China Second University Hospital, University, Chengdu, Sichuan, China

**Keywords:** pulmonary hypertension, statins, transcription factor, vascular remodeling

## Abstract

Vascular smooth muscle cell proliferation has been known to be predominant in vascular remodeling of pulmonary hypertensive. The GATA family proteins, a group of zinc finger transcription factors, play an important role during cell proliferation. The aim of present study was to investigate the expression of GATA-6 gene in experimental pulmonary hypertensive rats and explore the effect of regulation of GATA-6 expression by simvastatin on pulmonary vascular remodeling. The male Sprague-Dawley rats model was established with receiving pneumonectomy and monocrotaline (MCT) administration. Right pulmonary artery remodeling in these animals was compared with untreated rats or rats receiving simvastatin. The level of GATA-6 mRNA and protein expression was detected by reverse transcriptase–polymerase chain reaction (RT-PCR) and Western blotting, respectively. Pneumonectomized, MCT-treated rats had significantly increased mean pulmonary arterial pressure (mPAP), RV/(LV + S) ratio (ratio of the right ventricular to left ventricular and septum weights), vascular occlusion scores (VOSs), and percent media wall thickness on day 35, all the indices were significantly decreased after simvastatin administration in these rats. The level of GATA-6 mRNA and protein were markedly decreased in these pneumonectomy and MCT-treated rats, and they were significantly up-regulated in these rats after receiving simvastatin. These results indicate that the development and progression of pulmonary hypertension is prevented by simvastatin by up-regulating GATA-6 expression in the lung tissue.

Hypertensive pulmonary vascular disease is characterized by pulmonary vascular remodeling, involving abnormal proliferation of vascular endothelial and smooth muscle cells and extracellular matrix deposition, leading to occlusion of pulmonary arterioles, pulmonary hypertension, right ventricular failure, and death. Pulmonary arterial endothelial cell injury are considered prerequisites for the induction of advanced vascular remodeling in pulmonary arterial hypertension [1–4]. Migration, proliferation, and matrix synthesis of pulmonary artery smooth muscle cells play a key role in pulmonary vascular remodeling. Normal adult pulmonary arterial smooth muscle cells do not migrate, grow, or synthesize matrix once normal development is completed. Dysfunction of endothelium and elevated shear stress may result in smooth muscle cells proliferation, migration, and matrix synthesis. Increase in shear stress due to high flow modulates production of platelet-derived growth factor, transforming growth factor-β, nitric oxide, and transcription factors of the GATA family that regulate cell growth, migration, and matrix synthesis [5, 6].

The GATA family proteins are a group of zinc finger transcription factors that play an important role during mammalian organ morphogenesis, cell proliferation, and sex differentiation [7]. These factors bind to a consensus DNA motif (A/T)GATA(A/G) in the promoters of target genes in a variety of tissues [7]. Six GATA family members have been identified in vertebrates and can be divided into 2 groups based on their tissue distribution and homology: GATA-1, -2, and -3 and GATA-4, -5, and -6 [8]. GATA-1, -2, and -3 genes are predominantly expressed in hematopoietic cells where they are involved in proliferation and differentiation of several cell lineages [9–12]. GATA-4, -5, and -6 are expressed in heart, liver, lungs, and the gastrointestinal tract where they mediate tissue-specific gene expression, and GATA-6 is the only member of the GATA family expressed in vascular smooth muscle cells (VSMCs) [13–17].

Analysis of vascular smooth muscle cells has demonstrated an important role of GATA-6 in regulating cell proliferation in response to mitogenic or mechanical stimulation. GATA-6 mRNA levels are down-regulated in proliferating vascular smooth muscle cells, suggesting that GATA-6 expression is linked to the cell cycle in these cells and plays as an important regulator of the VSMC phenotype [18–20]. More specifically, forced expression of GATA-6 in vascular smooth muscle cells induces growth arrest through a mechanism involving enhanced expression of the cyclin-dependent kinase inhibitor p21 [20, 21]. In vivo, adenovirus-mediated gene transfer of GATA-6 in balloon-injured carotid arteries prevented vessel lesions associated with vascular smooth muscle cell phenotypic modulation [21]. Interestingly, laminar shear stress was recently demonstrated to activate transcription factor GATA-6 [22], which binds to a GATA consensus element located in the region of downstream gene promoter regulating these gene expression. A study in vitro reveals that GATA-6 regulates a set of genes associated with synthetic SMC functions and suggests that this transcriptional pathway may be independent from myocardin-induced SMC differentiation in VSMCs. An unbiased microarray screen of genes regulated by GATA-6 in VSMCs identifies that endothelin-1 and angiotensin 1a (AT1a) receptor genes are direct GATA-6 target genes [23]. Based on these studies, we speculate that activity of transcription factor GATA-6 may be regulated by disturbed laminar shear stresses occurring in pulmonary hypertension.

The 3-hydroxymethyl-3-methylglutaryl-coenzyme A (HMG-CoA) reductase inhibitors, statins, improve cardiovascular outcomes independent of their effects on cholesterol reduction [24]. The immunosuppressive and anti-inflammatory properties of statins [25] may contribute to the improved survival of patients with atherosclerosis [24, 26]. Statins can suppress endothelial and vascular smooth muscle cell proliferative and inflammatory responses to injury [27, 28]. These effects involve inhibition of isoprenylation of Rho and Rac family GTPases that couple growth factor receptors to the intracellular mitogen-activated protein/extracellular signal-regulated kinase (MAP/ERK) kinase signaling pathways and induction of the cell cycle inhibitor p27^*Kip*1^ [27, 28]. Statins also improve endothelium-dependent relaxation through mechanisms that involve induction of endothelial nitric oxide synthase (eNOS) and nitric oxide production [29, 30]. Several studies demonstrate that simvastatin, a member of the statin family, attenuates pulmonary vascular remodeling in rat neointimal and hypoxic models of pulmonary arterial hypertension (PAH) [31–33]. Interestingly, simvastatin is recently demonstrated in vitro to induce the expression of smooth muscle α-actin (SM-a-actin) and major histocompatibility complex (SM-MHC), highly specific markers of differentiated phenotype [34]. So we speculated whether pleiotropic atheroprotective effects of HMG-CoA reductase inhibitors may be mediated by the expression of other members of GATA family that play an important role in cell proliferation.

In this study, we used an animal model of pulmonary hypertension by combination of monocrotaline (MCT) administration with pneumonectomy to investigate the efficacy of simvastatin and its mechanism of reversing established neointimal vascular occlusion and pulmonary hypertension. We showed that the expression of GATA-6 mRNA and protein was down-regulated in this pulmonary hypertension model, which was reversed by simvastatin administration.

## MATERIALS AND METHODS

### Animal Model

Pathogen-free male Sprague-Dawley rats weighing between 300 and 400 g (12 weeks old) were used for this study. All animals were obtained from Sichuan University Animal Centre (Sichuan, Chendu, China) and received humane care. On day 0, rats were anesthetized by an intraperitoneal injection of 10% chloral hydrate (400 mg/kg) and injected subcutaneously atropine sulfate (50 μg/kg) in the right hindlimb. Left pneumonectomy was performed as previously described [35]. On day 7, rats were injected subcutaneously in the nape with monocrotaline (MCT) (60 mg/kg; Sigma, USA). MCT was dissolved in 0.5 N HCl and adjusted to pH 7.4 with 0.5 N NaOH solution. Rats were housed with a 12/12-hour light/dark cycle and given water and standard rat chow ad libitum.

### Treatment Groups

Pneumonectomized rats were randomized to receive simvastatin or vehicle by daily gavage, or neither simvastatin nor vehicle. Six groups were studied: Rats in group PMV_1–35_ (*n* = 12) received a vehicle from days 1 to 35. Group PMV_21–35_ (*n* = 12) received a vehicle from days 21 to 35. Group PMS_1–35_ (*n* = 12) received simvastatin (2 mg/kg per day) [31] from days 1 to 35. Group PMS_21–35_ (*n* = 12) received simvastatin (2 mg/kg per day) from days 21 to 35. Groups PM_1–21_ (*n* = 10) and PM_1–35_ (*n* = 12) received neither simvastatin nor vehicle, and were sacrificed on day 21 or 35 (post pneumonectomy) to provide reference point for disease progression in this model. Ten additional rats were studied as a control group without any intervention. On day 35 after pneumonectomy, rats were sacrificed and organs harvested for the following analysis.

### Hemodynamic Studies and Tissue Preparation

On day 35, rats were anesthetized with an intraperitoneal injection of 10% chloral hydrate. Mean pulmonary arterial pressures (mPAPs) were measured as previously described [36, 37]. After exsanguination, the right lung, right ventricle, left ventricle, and septum were collected for histology [36, 37]. Tissues were fixed in 10% neutral-buffered formalin, paraffin embedded, and sectioned. After EVG (elastin-van Gieson) staining, lung sections were examined histologically for evidence of pulmonary vascular disease. The severity of pulmonary vascular neointimal formation was assessed in 50 panacinar arteries from each animal. The severity of neointimal formation was expressed as the vascular occlusion score (VOS), which was scored according to the criteria of Okada and coworkers [35]. Briefly, the absence of neointimal formation or luminal occlusion equals grade 0; the presence of neointimal formation causing less than 50% luminal occlusion equals grade 1; the presence of neointimal formation causing greater than 50% luminal occlusion equals grade 2. An average score for 50 vessels (bounded by 0 and 2) was calculated for each animal. Samples of right lung were immediately placed in liquid nitrogen for extraction of total RNA.

### Pulmonary Artery Morphometry

Analysis of each section was carried out in a blinded fashion. To assess the degree of medial thickening of muscular pulmonary arteries, images of 30 to 50 vessels were recorded in subsets of animals at × 400. Each artery was classified by the structure of the accompanying airway as terminal bronchiole, respiratory bronchiole, alveolar duct, or alveolar wall. The software Image-Pro plus, version 4.5.0.29, was used to measure mean arterial diameter (between external elastic laminae) and media thickness (between internal and external elastic laminae) in complete muscular arteries that accompanied terminal and respiratory bronchioles. The percent medial wall thickness (%WT) was expressed as %WT = (media thickness × 2)/external diameter × 100 [38].

### GATA-6 Gene Expression Analysis

#### RNA Isolate and RT-PCR

Total RNA from rat lung was isolated using Trnzol (Tiantgen, China). Reverse transcriptase–polymerase chain reaction (RT-PCR) was used to amplify portions of the rat GATA-6 gene (GATA-6; GenBank accession number NM019185) and β-actin (β-actin; GenBank accession number NM031144) from rat lung. The primers used were

GATA-6, forward: 5′-CCCAGCGCAGACCTGTTGGAGGACC
GATA-6, reverse: 5′-TGTGACAGTTGGCACAGGACAG
β–Actin, forward: 5′-GACCCAGATCATGTTTGAGACC
β–Actin, reverse: 5′-GCAGTAATCTCCTCCTGCATCC

Reverse transcription was carried out with 1 μg of total RNA in a reaction volume of 20 μL using ReverTra Ace-a kit (ToYoBo) following the provided instructions. Following the reverse transcription of RNA template, 1 μL of the synthesized cDNA was amplified by PCR. The cycling parameters for each primer pair were analyzed independently in preliminary experiments. The PCR for GATA-6 was conducted at 95°C for 2 minutes, 95°C for 30 seconds, 61°C for 45 seconds, 72°C for 45 seconds for 33 cycles, followed by an additional 72°C 10 minutes for elongation in the final cycle. The PCR for β-actin was conducted at 94°C for 3 minutes, 94°C for 30 seconds, 61°C for 30 seconds, 72°C for 1 minutes for 30 cycles, followed by an additional 72°C 5 minutes for elongation in the final cycle. Finally, the RT-PCR product was analyzed by gel electrophoresis in a 1% agarose gel, stained by ethidium bromide (Sigma, USA) and then visualized under UV (ultraviolet) light. Gel electrophoretic analysis was performed with a UVP GelWorks system (1D Intermediate Version 3.01; USA) to quantify the relative levels of GATA-6 to β-actin.

#### Western Blot Analysis

Whole-cell lysates were prepared and Western blot analyses were performed to detect GATA-6 protein expression. In brief, 100 mg of lung tissue was homogenated with 1 ml of tissue protein lysis buffer (MT-CelLytics; Biocolors, China). Equivalent amounts of protein (100 μg) were separated by 10% sodium dedecyl sulfate-polyacrylamide gel electrophoresis (SDS-PAGE) minigels for approximately 100 minutes at 200 V to separate the region between 50 and 60 kDa and were electroblotted onto polyvinylidene difluoride transfer membrane (Roche, Germany). Blocked membranes were incubated with polyclonal antibody GATA-6 (Santa Cruz; 1:750) in Tris-buffered saline supplemented with 0.05% of Tween-20 (TTBS) plus 1% nonfat milk overnight at 4°C. The GATA-6 antibody recognizes the first 20 amino acids at the C-terminus of recombinant human GATA-6. No blocking peptides are available for these antibodies. Following extensive washing with TTBS, membranes were incubated with a 1:1000 dilution of horseradish peroxidase–conjugated goat anti-rabbit immunoglobulin G (IgG) secondary antibodies (Santa Cruz; 1:1000) in TTBS plus 5% milk for 1 hour at room temperature. After extensive washing with TTBS, protein-antibody complexes were visualized by the enhanced chemiluminescene detection system (Pierce, USA). Membranes were reprobed for actin (Santa Cruz; 1:2000).

### Statistical Analysis

Data were expressed as means ± SD. One-way analysis of variance (ANOVA) was used to compare means between groups and the Newman-Keuls method for post hoc multiple comparisons assuming unequal variances using SPSS13.0 software. A *P* value of <.05 was taken to indicate that conventional statistical significance had been achieved.

## RESULTS

### Simvastatin Prevented Development of Pulmonary Arterial Hypertension

**FIGURE 1:**
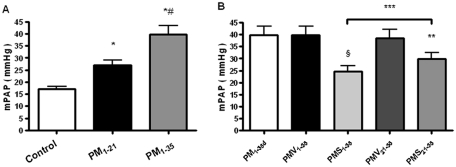
Simvastatin prevents the development and progression of pulmonary arterial hypertension in pneumonectomized, MCT-treated rats. (*A*) Mean pulmonary arterial pressures (mPAPs) in group PM_1–21_ (pneumonectomized, MCT-treated rats that were sacrificed on day 21; *n* = 10), Group PM_1–35_ (pneumonectomized, MCT-treated rats that were sacrificed on day 35; *n* = 8), and control (normal rats; *n* = 10). Bars are means ± SD. *P < .001 for groups PM_1–21_ and PM_1–35_ versus control; ^#^*P* < .001 for group PM_1–21_ versus group PM_1–35_. (*B*) Mean pulmonary arterial pressures (mPAPs) in group PM_1–35_ as stated in *A*, group PMV_1–35_ (MCT-treated, pneumonectomized rats that received vehicle from days 1 to 35; *n* = 9), group PMS_1–35_ (MCT-treated, pneumonectomized rats that received simvastatin [2 mg/kg per day] from days 1 to 35; *n* = 12), group PMV_21–35_ (MCT-treated, pneumonectomized rats that received vehicle from days 21 to 35; *n* = 8), group PMS_21–35_ (MCT-treated, pneumonectomized rats that received simvastatin [2 mg/kg per day] from days 21 to 35; *n* = 10). Bars are means ± SD. ^§^*P* < .001 for group PMS_1–35_ versus group PMV_1–35_; **P < .001 for group PMS_21–35_ versus group PMV_21–35_; ***P < .001 for Group PMS_1–35_ versus group PMS_21–35_.

Rats in group PM_1–21_ gradually developed severe pulmonary arterial hypertension (PAH) by day 21 (Figure 1*A*). Four out of 12 rats in group PM_1–35_ died by day 35 and the remainder demonstrated progression to severe PAH (Figure 1*A*).

All simvastatin-treated rats in group PMS_1–35_ survived the entire course of the study (35 days). Two of 12 rats in group PMS_21–35_ died by day 35. Simvastatin delayed or partly reversed PAH and decreased mPAP in group PMS_1–35_ or PMS_21–35_, compared with the vehicle-treated rats in group PMV_1–35_ or PMV_21–35_ on day 35, respectively (Figure 1*B*).

### Right Ventricular Hypertrophy

**FIGURE 2:**
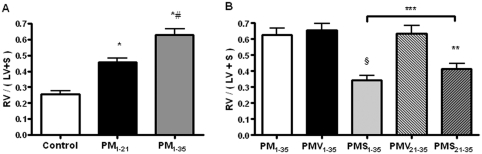
Simvastatin prevents the development and progression of right ventricular hypertrophy in pneumonectomized, MCT-treated rats. (*A*) Ratios of right vertricular weight to left ventricular and inter-ventricular septal weights [RV/(LV + S)] are shown among the 3 groups of rats as stated in Figure 1*A*. Bars are means ± SD. *P < .001 for groups PM_1–21_ and PM_1–35_ versus control; ^#^*P* < .001 for group PM_1–35_ versus group PM_1–21_. (*B*) Ratios of RV/(LV + S) are shown for the 5 groups of rats as stated in Figure 1*B*. Bars are means ± SD. ^§^*P* < .001 for group PMS_1–35_ versus group PMV_1–35_; **P < .001 for group PMS_21–35_ versus group PMV_21–35_; ***P < .001 for group PMS_21–35_ versus group PM_1–35_.

By day 35, pulmonary hypertensive rats demonstrated significant right ventricular hypertrophy. The ratio of the right ventricular to left ventricular and septum weights, RV/(LV + S), of pulmonary hypertensive rats significantly increased in comparison with normal rats (Figure 2*A*).

Simvastatin prevented progression and partly reversed established right ventricular hypertrophy of diseased rats on day 35, with groups PMS_1–35_ and PMS_21–35_ showing lower RV/(LV + S) ratio (Figure 2*B*) compared with groups PMV_1–35_ and PMV_21–35_, respectively (Figure 2*B*).

### Simvastatin Decreased Medial Hypertrophy

**FIGURE 3:**
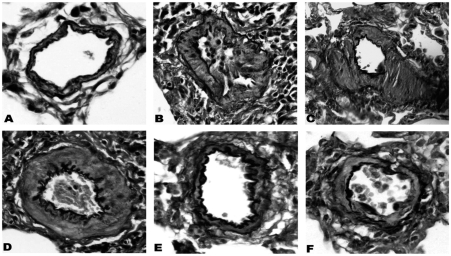
Simvastatin prevents the development of small pulmonary artery hypertrophy in pneumonectomized, MCT-treated rats. (*A*) Control; (*B*) PMV_1–35_; (*C*) PMV_21–35_; (*D*) PM_1–35_; (*E*) PMS_1–35_; (*F*) PMS_21–35_. Elastin-van Gieson staining (magnification, × 200) images show reduced small pulmonary artery hypertrophy in Groups PMS_1–35_ and PMS_21–35_.

**FIGURE 4:**
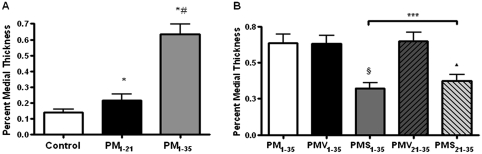
Reduced percent medial wall thickness of small pulmonary artery hypertrophy in pneumonectomized, MCT-treated rats by simvastatin. Small pulmonary artery (50 to 150 μm) hypertrophy measured by (2 × medial thickness/external diameter) × 100. (*A*) Percent medial wall thickness is shown among the 3 groups of rats as stated in Figure 1*A*. Bars are means ± SD. *P < .001 for Groups PM_1–21_ and PM_1–35_ versus control; ^#^*P* < .001 for group PM_1–35_ versus group PM_1–21_. (*B*) Percent medial wall thickness is shown among the 5 groups of rats as stated in Figure 1*B*. Bars are means ± SD. Simvastatin alleviated arterial medial hypertrophy in pneumonectomized, monocrotaline-treated rats (^§^*P* < .001 for group PMS_1–35_ versus group PMV_1–35_; ^▴^*P* < .001 for group PMS_21–35_ versus group PMV_21–35_). Group PMS_1–35_ had significantly lower percent medial wall thickness than group PMS_21–35_ (***P < .05).

Prominent medial wall hypertrophy was evident in muscular pulmonary arteries from diseased rats (Figure 3*A* to *F*). The percent medial wall thickness (%WT) of rats in groups PM_1–21_ and PM_1–35_ was significantly higher than that of respective control animals (Figure 4*A*).

In contrast, rats treated with simvastatin (groups PMS_1–35_ and PMS_21–35_) demonstrated decreased medial hypertrophy at different degree (Figure 4*B*), whereas rats treated with vehicle (groups PMV_21–35_ and PMV_1–35_) demonstrated significant medial hypertrophy (Figure 4*B*).

### Histopathology

**FIGURE 5:**
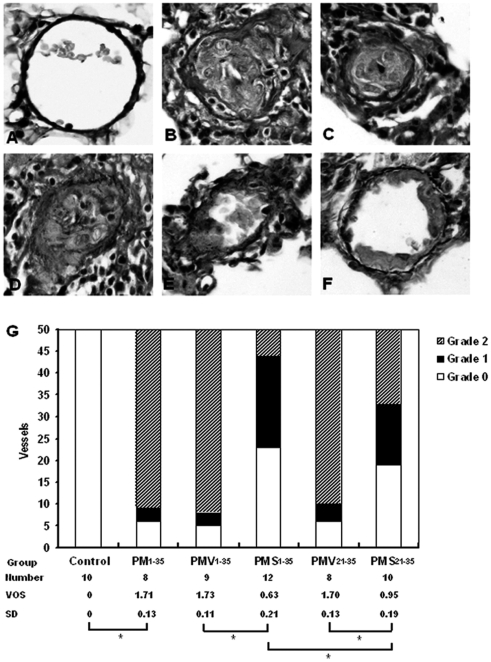
Simvastatin suppresses the development of pulmonary arterial neointimal formation. The vascular occlusion score (VOS) was the average of 50 consecutive intra-acinar pulmonary arteries. (*A*) Normal rat intra-acinar artery without evidence of neointimal proliferation (grade 0). (*B*, *C*, and *D*) Grade 2 neointimal lesions (>50% luminal occlusion) in groups PM_1–35_, PMV_1–35_, and PMV_21–35_. (*E*) Predominance of grade 2 lesson in group PMS_21–35_. (*F*) Grade 1 neointimal lesion (<50% lumenal occlusion) in group PMS_1–35_. All samples were stained with EVG. The images are × 400 magnification. (*G*) Average grades of VOS among the 6 groups of rats as stated in Figure 1*A* and *B*. *P < .001 for group PM_1–35_ versus control; *P < .001 for group PMS_1–35_ versus group PMV_1–35_; *P < .001 for group PMS_21–35_ versus group PMV_21–35_; *P < .001 for group PMS_1–35_ versus group PMS_21–35_, respectively.

Representative morphologies of small pulmonary arteries in normal rats and rats in groups PM_1–35_, PMV_1–35_, PMV_21–35_, PMS_1–35_, and PMS_21–35_ are shown to be stained for elastin-van Gieson to reveal the inner elastin lamina (Figure 5*A* to *F*). A quantitative analysis of luminal obstruction on 50 consecutive small pulmonary arteries from each rat was performed (Figure 5*G*). The distribution of the vascular lesions, and an average vascular occlusion score (between 0 and 2), are presented (Figure 5*A* to *F*). The pneumonectomized, MCT-treated rats resulted in severe changes of neointimal proliferation and vascular occlusion (Figure 5*B* versus *A*). The vascular occlusion score (VOS) in groups PMV_1–35_ and PMV_21–35_ was similar to an average VOS for our laboratory's historical data on group PM_1–35_ rats (Figure 5*C* and *D* versus *B*). This suggests that the treatment of vehicle does not alter the histopathology of this disease model. Compared with groups PMV_1–35_ and PMV_21–35_, groups PMS_1–35_ and PMS_21–35_ had a lower VOS, respectively (Figure 5*G*).

### GATA-6 Gene Expression in the Lung Tissue

**FIGURE 6:**
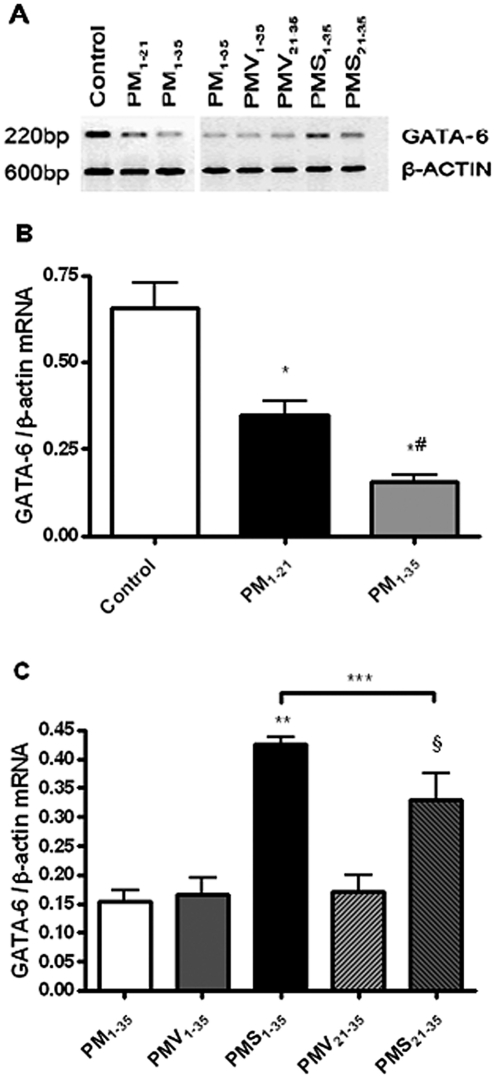
Reduction in GATA-6 mRNA expression during the development of pulmonary hypertension in pneumonectomized, MCT-treated rats that was reversed by simvastatin. (*A*) RT-PCR analysis of GATA-6 and β-actin gene expression in representative rat lungs from groups stated in Figure 1*A* and *B*. (*B*) Relative abundance of GATA-6/β-actin mRNA expression among the 3 groups of rats stated in Figure 1*A*. Mean and standard deviation of GATA-6 mRNA expression normalized to β-actin is shown (*n* = 3/group). *P < .001 for groups PM_1–21_ and PM_1–35_ versus control; ^#^*P* < .01 for group PM_1–35_ versus group PM_1–21_. (*C*) Relative abundance of GATA-6/β-actin mRNA expression among the 5 groups of rats stated in Figure 1*B*. Mean and standard deviation of GATA-6 mRNA expression normalized to β-actin is shown (*n* = 3/group). Simvastatin restored GATA6 mRNA expression level in diseased rats (**P < .001 for group PMS_1–35_ versus group PMV_1–35_; ^§^*P* < .001 for group PMS_21–35_ versus group PMV_21–35_). Group PMS_1–35_ had significantly higher GATA-6 mRNA expression level than Group PMS_21–35_ (***P < .01).

Changes in lung GATA-6 mRNA expression were assessed semiquantitatively by RT-PCR (Figure 6*A*). The expression of GATA-6 mRNA, normalized to β-actin expression, was significantly lower in diseased rats (groups PM_1–21_ and PM_1–35_) than in normal rats (Figure 6*B*). Simvastatin treatment increased the expression of GATA-6 mRNA in MCT-treated, pneumonectomized rats (Figure 6*C*).

**FIGURE 7:**
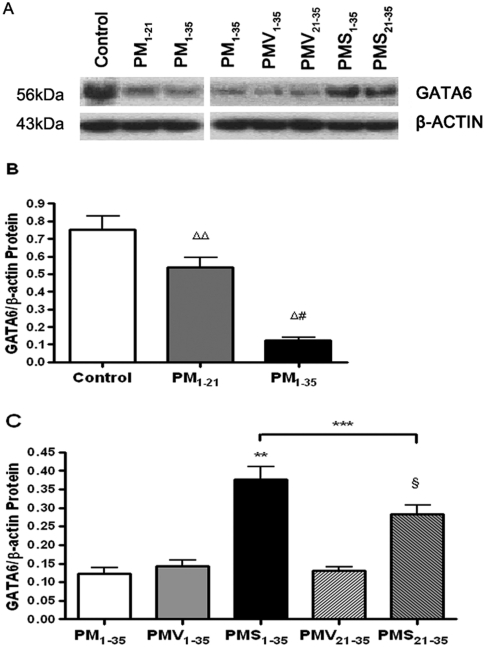
Decrease in GATA-6 protein expression during the development of pulmonary hypertension in pneumonectomized, MCT-treated rats that was restored by simvastatin. (*A*) Western blotting analysis of GATA-6 protein expression in representative rat lung protein extracts from each group as stated in Figure 1*A* and *B*. (*B*) Relative abundance of GATA-6/β-actin protein expression among the three groups of rats stated in Figure 1*A*. Mean and standard deviation of GATA-6 protein expression normalized to β-actin is shown (*n* = 3/group). *P < .001 for group PM_1–35_ versus control; **P < .01 for group PM_1–21_ versus control; ^#^*P* < .01 for group PM_1–35_ versus group PM_1–21_. (*C*) Relative abundance of GATA-6/β-actin protein expression among the 5 groups of rats stated in Figure 1*B*. Mean and standard deviation of GATA-6 protein expression normalized to β-actin is shown (*n* = 3/group). Simvastatin restored GATA-6 protein expression level in diseased rats (***P < .001 for group PMV_1–35_ versus group PMS_1–35_; ^§^*P* < .001 for group PMV_21–35_ versus group PMS_21–35_). Group PMS_1–35_ had significantly higher GATA-6 protein expression level than group PMS_21–35_ (****P < .01).

The lung expression of GATA-6 protein was assessed qualitatively by Western blotting (Figure 7*A*). A GATA-6 antiserum-immunoreactive band is detected at 56 kDa (Figure 7*A*). Compared with normal rats, diseased rats in groups PM_1–21_ and PM_1–35_ showed decreased expression of GATA-6 protein (Figure 7*B*). Simvastatin treatment significantly increased GATA-6 protein expression (Figure 7*C*).

## DISCUSSION

Pulmonary arterial hypertension is characterized histopathologically by changes of abnormal proliferation of vascular endothelial and smooth muscle cells, occlusion of microvascular pulmonary arteries, and plexiform lesion formation in severe cases. Although the pathogenesis of most forms of pulmonary arterial hypertension is unknown, there have been many recent developments in pathogenesis involving inappropriate proliferation and constriction of vascular smooth muscle cells, and deficiencies of endogenous vasodilators such as prostacyclin and endothelial-derived nitric oxide [39], especially pertaining to the molecular genetics and cell biology of idiopathic pulmonary arterial hypertension. The increase in pulmonary arterial pressure leads to right ventricular congestive failure and ultimately to death. Current treatment strategies for PAH include diuretics, anticoagulation, digoxin, and supplemental oxygen for congestive heart failure, and pulmonary vasodilating agents such as calcium channel antagonists and prostanoids, especially intravenous (IV) epoprostenol and endothelin-receptor antagonists. New therapies, especially theose directing at suppressing inappropriate neointimal proliferation in pulmonary arteries, are warranted [40–43].

Statins, the HMG CoA reductase inhibitors, have had a dramatic impact on clinical outcomes in patients with coronary artery disease. Several studies have documented benefits unrelated to cholesterol lowering [44, 45], with multiple “pleiotropic” effects on vascular wall function that would be expected to attenuate vascular remodeling [46]. These effects stem from their ability to reduce the production of the isoprenoid intermediates farnesyl and geranylgeranyl pyrophosphate, compounds that are distal to mevalonate in the cholesterol synthetic pathway. These lipophilic molecules are then covalently bound to Rho and other small G proteins in a post-translational modification that is essential for attachment of these important signaling proteins to cell membranes and regulators and for their activation of downstream effectors [47].

In present study, we probed the hypothesis that statins could attenuate vascular remodeling and pulmonary hypertension in a rat model of neointimal pulmonary hypertension. Simvastatin administration produced a marked reduction in pulmonary artery pressure developed in animals with pneumonectomy and MCT administration. Simvastatin also significantly reduced the right ventricular hypertrophy, as indicated by the RV/(LV + S) weight ratio. Morphological analysis of the pulmonary vasculature of simvastatin-treated rats revealed considerable decrease in medial thickness of medium-sized arteries and neointimal formation. We also found that GATA-6 mRNA and protein expression dramatically decreased in pneumonectomized, MCT-treated rats that was restored by simvastatin administration.

The exact mechanism of attenuation of neointimal pulmonary hypertension by simvastatin was not understood. Several actions of statins could account for our observations. Statins have been noted to reduce blood pressure in spontaneously hypertensive but not normotensive rats [38]. Simvastatin treatment potently inhibits vascular remodeling by attenuates chronic hypoxic pulmonary hypertension and polycythemia in rats [33], and reverses pulmonary arterial neointimal formation and PAH after toxic injury by down-regulating the inflammatory genes fos, jun, and tumor necrosis factor-α and up-regulating the cell cycle inhibitor p27Kip1, endothelial nitric oxide synthase, and bone morphogenetic protein receptor type 1a [31]. Inhibition of HMG-CoA reductase by statins decreases isoprenoid intermediates such as farnesyl-PP and geranylgeranyl-PP, which leads to an inhibition of isoprenylation of small GTPases such as Ras, Rho, Rab, and Rap. Among the Rho GTPases are RhoA, Rac1, and Cdc42 [48]. Statins have been shown to induce apoptosis of pulmonary vascular smooth muscle cells in serum-free medium [49]. Both endothelin-1 [50] and endothelin receptor [51] transcriptions are reduced by statins, which would attenuate chronic hypoxic pulmonary hypertension. Simvastatin markedly decreased the platelet-derived growth factor–induced proliferation of vascular smooth muscle cells by preventing Rho GTPase-induced downregulation of p27^kip1^, an important negative regulator of cell proliferation [28]. Simvastatin is also demonstrated to regulate the differentiation and the phenotype expression of VSMCs [34]. In addition, statins have potent antioxidant effects [52]. Given that oxidative stress may be an important mediator of chronic hypoxic pulmonary hypertension [38] as well as acute hypoxic pulmonary vasoconstriction [53], the antioxidant effect of simvastatin may also play a role in attenuation of pulmonary hypertension observed here.

In this study, we found that down-regulation of GATA-6 expression was concurrent with development and progression of pulmonary hypertension, right ventricular hypertrophy, arterial medial hypertrophy, and neointimal formation in the neointimal pulmonary hypertensive model. GATA-6 gene expression has been detected in the pericardial mesoderm, embryonic heart tube, and primitive gut during early embryonic development [54]. However, during development, GATA-6 becomes the only member of the family expressed in vascular smooth muscle cells [15, 55] and its expression is down-regulated in proliferating vascular smooth muscle cells [18]. These data suggest that down-regulation of GATA-6 expression is closely associated with pulmonary arterial smooth muscle cells proliferation and that the level of GATA-6 expression may be an indicator of severity of hypertensive pulmonary vascular disease.

Some recent reports show that simvastatin up-regulates GATA-3 expression in dendritic cells and up-regulates the binding activity of GATA-6 to SM-MHC GATA site, whereas mutating the GATA-6 binding site abolished this activation [34, 56]. In the present study, we also found that down-regulation of GATA-6 expression was reversed in pulmonary hypertensive rats treated with simvastatin. However, the precise mechanism that statins regulate expression of GATA family members is unknown. Further studies will be performed to investigate the mechanism of GATA-6 expression by regulated by simastatin and examine GATA-6 expression in the lung tissue of human patients with pulmonary hypertension.
